# A Benchmark Corpus of Yemeni Proverbs for Figurative and Cultural Language Modeling

**DOI:** 10.1038/s41597-026-07234-y

**Published:** 2026-05-11

**Authors:** Nasser Thmer, Ali Al-Laith, Muhammad Shoaib, Hassan Alhuzali

**Affiliations:** 1 Computer Science Department, University of Engineering and Technology Lahore, Lahore, Pakistan; 2 Department of Computer, College of Education Lawder, University of Abyan, Abyan, Yemen; 3 Nordic Studies and Linguistics Department, University of Copenhagen, Copenhagen, Denmark; 4 Department of Computer Science and Artificial Intelligence, Umm Al-Qura University, Makkah, Saudi Arabia

**Keywords:** Scientific data, Computer science

## Abstract

We present a structured corpus of 5,252 Yemeni Arabic proverbs paired with explanations in Modern Standard Arabic (MSA). The dataset was compiled from four printed proverb anthologies and three publicly accessible online repositories between January and June 2024. All entries were manually transcribed or programmatically extracted and subsequently verified to ensure accuracy and fidelity to the original sources. Each record includes the proverb text, its explanation, source attribution, and available geographic metadata. The corpus addresses the scarcity of dialect-specific Arabic resources and provides a benchmark for evaluating figurative language understanding and culturally grounded language generation. Technical validation through supervised fine-tuning and unsupervised clustering demonstrates the dataset’s internal consistency and thematic diversity. The dataset is publicly available via Zenodo under a Creative Commons Attribution 4.0 International (CC BY 4.0) license.

## Background & Summary

Proverbs encapsulate cultural wisdom and societal values, offering rich insights into a community’s linguistic creativity and metaphorical expression. However, their figurative and context-dependent nature poses significant challenges for NLP systems, particularly in low-resource languages such as Yemeni Arabic. Although prior work has explored proverb processing in widely studied languages (e.g., English, Mandarin)^1^, resources for Arabic dialects, especially Yemeni, remain scarce. This gap limits the development of culturally-aware NLP tools capable of handling nuanced, non-Western linguistic phenomena.

Recent advances in LLMs have demonstrated remarkable capabilities in understanding figurative language^1,2^. However, their performance on culturally nuanced tasks is constrained by dependencies on training data and prompting strategies. Benchmarks like ePiC^3^, Jawaher^4^, and Absher^5^ have laid groundwork for evaluating proverb interpretation, but they lack coverage of Yemeni dialects. Ethnographic studies^6,7^ and translation research^8,9^ further underscore the need for computational resources that preserve cultural specificity.

To address these gaps, we present a meticulously curated dataset of Yemeni proverbs sourced from printed materials^10–13^ and online repositories. The dataset underwent manual transcription and verification to mitigate challenges such as Optical Character Recognition (OCR) inaccuracies and dialectal variations. Each proverb is paired with an expert-annotated explanation, ensuring fidelity to cultural and linguistic context.

This dataset serves as a benchmark for evaluating LLMs’ ability to interpret culturally grounded language. Our preliminary experiments with models like GPT-4o^14^, Gemini 1.5 Pro, and ALLaM-7B^15^ reveal the impact of few-shot prompting and model scale on performance, while also exposing limitations in cultural sensitivity. By making this resource publicly available, we aim to advance research in low-resource NLP, cross-cultural communication, and figurative language understanding. This work aligns with broader efforts to diversify NLP resources^16,17^ and underscores the importance of culturally-informed model evaluation. Future applications include machine translation, educational tools, and anthropological research.

## Methods

### Sources and Selection Criteria

The dataset was compiled from seven primary sources selected based on: (1) recognized authority in Yemeni folkloric literature, (2) geographic diversity, and (3) accessibility for verification.

Four printed and digitalized proverb anthologies were used:

- *Al-Tharwah Al-Yamaniyyah min Al-Amthal Al-Shabiyyah*, Mohammed Othman Al-Adimi^11^.
- *Qutoof min Al-Amthal Al-Yamaniyyah*, Ahmed Mahdi Salem^13^.
- *Mujam Al-Amthal Al-Yamaniyyah Al-Shaiah*, Ahmed Ali Al-Hamdani^10^.
- *Al-Shai min Amthal Ahl Yafi*, Ali Saleh Al-Khulaki^12^.

Three online repositories were also included:

- *Folk Culture Magazine* — “Proverbs from Ibb” section^18^.
- *Abu Saeed Al-Abbasi Forum* — Hadhrami Proverbs^19^.
- *Old Sanaa Website* — Encyclopedia of Yemeni Proverbs^20^.

Digital sources were accessed between January and June 2024.

### Transcription and Digitization

All printed materials were manually transcribed by the first author, a native speaker of Yemeni Arabic. Manual transcription was chosen to avoid OCR errors and preserve dialectal orthography. Page numbers were recorded for internal verification.

Online sources were retrieved using Python (v3.10) with the requests and BeautifulSoup4 libraries. Extracted entries were manually reviewed to confirm correct pairing of proverbs and explanations and to remove HTML artifacts.

All entries were consolidated into structured spreadsheets before further processing.

### Data Cleaning and Normalization

Duplicate entries were identified using exact string matching on the proverb field, resulting in the removal of 77 records. Seventeen entries with incomplete or placeholder explanations were excluded.

Normalization included:

- Removal of Arabic diacritics
- Standardization of punctuation and whitespace
- Unification of alif variants
- UTF-8 encoding validation

No geographic labels were inferred when not explicitly stated in the source material.

### Verification and Quality Control

Each entry underwent two-stage verification:

1. Cross-check during transcription or extraction.
2. Full dataset review after consolidation.

All proverb-explanation pairs were manually confirmed to ensure accuracy and completeness. No new explanatory annotations were authored; explanations were transcribed directly from the source materials.

### Schema Design

The finalized dataset contains six fields:

- **id**: Unique integer identifier.
- **proverb**: Dialectal Yemeni proverb (UTF-8).
- **explanation**: Explanation in Modern Standard Arabic.
- **source**: Source title or platform name.
- **city**: Geographic origin if specified; otherwise null.
- **url**: URL for online sources; null for printed sources.

### Licensing and Redistribution

The original printed and digital sources do not consistently provide explicit open licenses. Therefore, no scanned pages or full source documents are redistributed.

The released dataset contains structured textual records and metadata derived from publicly accessible materials. The dataset itself is distributed under a Creative Commons Attribution 4.0 (CC BY 4.0) license. Users must consult original publishers for access to source documents under their respective terms.

## Data Records

The Yemeni Proverbs corpus is publicly available via a DOI-backed repository at Zenodo^21^. The dataset is distributed as a UTF-8 encoded JSON file entitled Yemeni_proverbs.json and contains 5,252 structured records.

Each record corresponds to a single proverb-explanation pair and includes source attribution and available geographic metadata. The dataset is released under a Creative Commons Attribution 4.0 (CC BY 4.0) license.

### File Format

The JSON file is structured as a list of objects. Each object contains the following fields:

- **id**: Unique integer identifier assigned sequentially.
- **proverb**: Dialectal Yemeni Arabic proverb in UTF-8 encoding.
- **explanation**: Explanation in Modern Standard Arabic (MSA), transcribed verbatim from the source material.
- **source**: Title of the printed anthology or name of the digital repository.
- **city**: Geographic origin when explicitly stated in the source; otherwise null.
- **url**: Direct link to the online source when applicable; null for printed sources.

### Example Record

An example entry from the dataset is presented in Table 1.

**Table 1:** Field-level schema of the Yemeni Proverbs Dataset.

### Dataset Composition

The final corpus contains 5,252 proverb-explanation pairs after removal of duplicate entries and incomplete records during preprocessing.

Geographic metadata is available for 1,420 entries (27%), covering multiple Yemeni regions including Ibb, Sana’a, Aden, and Yafi. The remaining entries do not specify regional origin in their source materials.

The dataset draws from seven primary sources. The distribution of entries by source is documented in Table 2.

**Table 2:** Distribution of Proverbs by Source.

Source	Count	Percentage (%)
Kitab: Al-Tharwah Al-Yamaniyyah min Al-Amthal Al-Sha’biyyah	2352	44.78
Kitab: Qutoof min Al-Amthal Al-Yamaniyyah	1007	19.17
Abu Saeed Al-Abbasi Forum	805	15.33
Kitab: Mu’jam Al-Amthal Al-Yamaniyyah Al-Sha’i’ah	552	10.51
Old Sanaa Website	229	4.36
Kitab: Al-Shae’i min Amthal Yafi’, Dr. Ali Saleh Al-Khulaki	174	3.31
Folk Culture Magazine	133	2.53

Table 3 provides a statistical overview of the dataset’s scale and coverage:

**Table 3:** Summary Statistics of the Yemeni Proverbs Dataset.

Statistic	Value
Total number of proverbs	5,252
Average proverb length (words)	4.87
Average explanation length (words)	16.91
Number of distinct sources	7
Largest single source (*Al-Tharwah*) contribution	44.78%

### Data Integrity

The JSON file was validated using Python’s built-in json module to ensure syntactic correctness and UTF-8 encoding integrity. All records were checked for balanced quotation marks, proper field closure, and consistent null formatting.

No scanned images or full copyrighted source documents are redistributed. The dataset contains structured textual records and metadata derived from publicly accessible materials. Users seeking access to original publications must consult the respective publishers or repositories.

## Technical Validation

This section builds upon the dataset structure and metadata described in Data Record Section, providing quantitative evidence of its quality and applicability.

### Model-based Usability Assessment

To assess the learnability and generalizability of the dataset in a supervised generative context, we fine-tuned the Arabic sequence-to-sequence model UBC-NLP/AraT5v2-base-1024^22^ using the Hugging Face Transformers library. The entire dataset was split into training (80%), validation (10%), and testing (10%) subsets. The training setup employed a learning rate of 5e-5, a linear scheduler with warmup ratio 0.1, num_train_epochs=20, and a batch size of 16 per device, with mixed-precision (FP16) optimization enabled. Early stopping was triggered at epoch 12 based on a stagnation in validation loss while training loss continued to decrease.

**Fig. 1:**
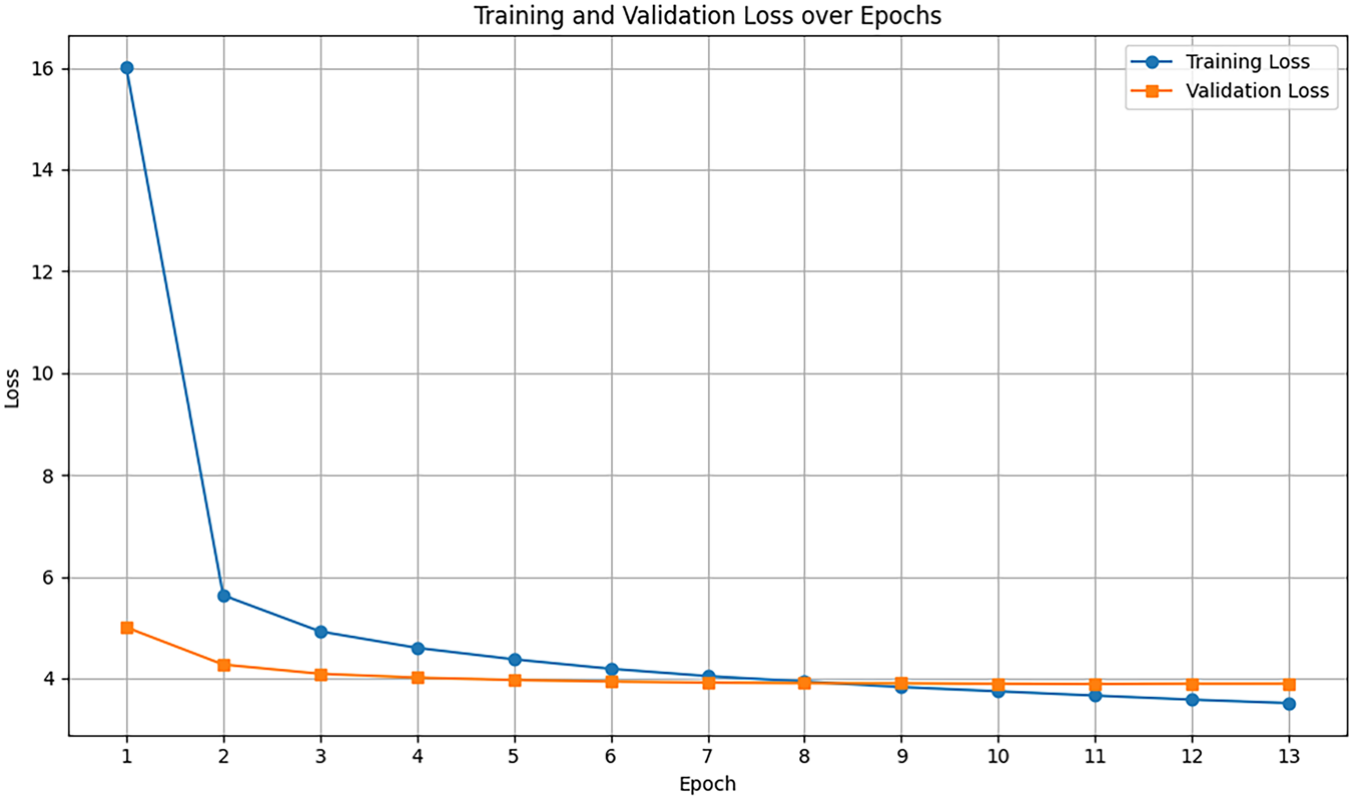
Training and validation loss over epochs during the fine-tuning of AraT5v2 on the Yemeni proverbs dataset. Early stopping was applied at epoch 12 to prevent overfitting.

As depicted in Fig. 1, training and validation loss decreased consistently during the initial epochs, with validation loss reaching a minimum around 3.89. This indicates that the dataset is well-suited for structured generation tasks.

To further evaluate the consistency and relevance of model-generated explanations, we performed automatic evaluation on the held-out test set (10% of the corpus) using a set of standard surface-level and semantic similarity metrics. These included BLEU^23^, ROUGE-1, ROUGE-2, and ROUGE-L^24^, chrF++^25^, and BERTScore-F1^26^. In addition, cosine similarity was computed between sentence embeddings derived from three pre-trained models: CAMeL-BERT^27^, MiniLM^28^, and Arabic-KW^29^. Finally, the SAS (Semantic Accuracy Score) metric^30^ was employed to measure conceptual alignment between reference and generated explanations. The resulting automatic evaluation scores are summarized in Table 4.

**Table 4:** Automatic evaluation metrics for generated explanations (averaged over the test set).

Metric	Score
BLEU	0.0213
ROUGE-1	0.1464
ROUGE-2	0.0314
ROUGE-L	0.1369
chrF++	15.4527
BERTScore-F1	0.7167
Cosine Similarity (CAMeL)	0.8086
Cosine Similarity (MiniLM)	0.7093
Cosine Similarity (Arabic-KW)	0.5092
SAS (SBERT-100K)	0.3515

All evaluations were conducted using the Hugging Face evaluate library, complemented by custom scripts for cosine similarity and SAS computations. The relatively low BLEU and ROUGE scores reflect the high lexical variability inherent in figurative explanation tasks, where multiple valid phrasings can convey the same meaning. In contrast, the higher BERTScore and cosine similarity values indicate strong semantic alignment between generated and reference explanations despite surface-level differences. Together, these findings confirm that the dataset is suitable for generative modeling while highlighting the intrinsic difficulty of culturally grounded proverb interpretation.

These results confirm that the dataset contains coherent, informative explanations suitable for generative modeling. The metrics suggest moderate to high semantic alignment between references and model outputs, despite the inherent difficulty of the task.

### Unsupervised Thematic Clustering

To evaluate the thematic diversity within the dataset, we performed unsupervised clustering using sentence-level embeddings derived from the Arabic-Triplet-Matryoshka-v2 model model^31^. Each proverb was encoded into a dense vector representation and then reduced to two dimensions using Uniform Manifold Approximation and Projection (UMAP)^32^ with cosine distance as the similarity metric. The resulting low-dimensional vectors were clustered using HDBSCAN^33^, configured with min_cluster_size=10 and Euclidean distance. The chosen cluster size parameter was determined empirically to balance thematic coherence and interpretability.

**Fig. 2:**
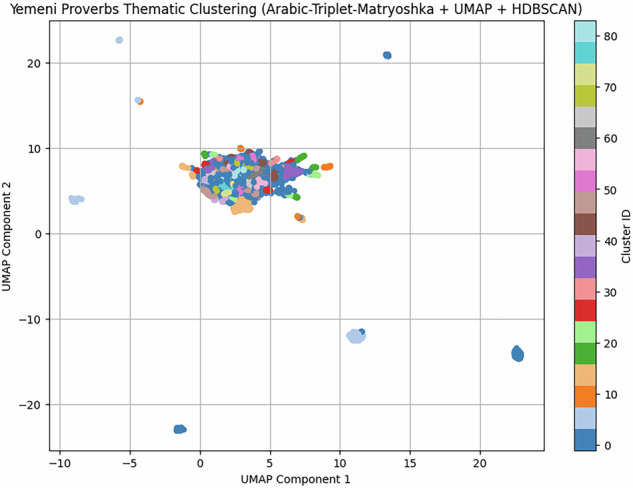
Thematic clustering of Yemeni proverbs using semantic embeddings from Arabic-Triplet-Matryoshka, projected using UMAP and grouped with HDBSCAN. Each point represents a proverb, colored by its assigned cluster.

This process yielded **84 distinct thematic clusters**. As commonly observed with HDBSCAN, some proverbs (assigned Cluster ID = -1) were labeled as *noise* due to insufficient density and thus excluded from structured groupings. Figure 2 presents a visualization of the clusters in 2D space. Table 5 lists representative clusters, their most salient keywords, and corresponding thematic interpretations.

**Table 5:** Selected clusters from the unsupervised thematic analysis with their top keywords and inferred topics.

The clustering results confirm that the dataset spans a broad spectrum of culturally embedded themes. The high topical purity observed within individual clusters suggests the utility of the dataset for downstream applications such as semantic clustering, figurative type classification, and sociolinguistic analysis.

### Supplementary Evaluation with Large Language Models (LLMs)

To further assess the quality and semantic richness of the dataset, we include results from a companion evaluation study involving seven prominent large language models (LLMs): GPT-4o, Gemini 1.5 Pro, ALLaM-7B, Jais-13B, LLaMA-3-8B, DeepSeek-7B, and Mistral-7B. These models were evaluated on the task of explaining 456 Yemeni proverbs under both zero-shot and few-shot prompting conditions.

We conducted automatic evaluation using Cosine Similarity, BERTScore, and Semantic Answer Similarity (SAS) with Arabic SBERT embeddings. In addition, a structured human evaluation was performed on the generated explanations using a 5-point Likert scale across three criteria: semantic accuracy, cultural appropriateness, and clarity. The human evaluation was conducted only on the zero-shot and few-shot generations, and not on the fine-tuned AraT5v2 model. The annotators were selected from distinct Yemeni dialect regions to ensure cultural grounding. Inter-annotator agreement measured by Krippendorff’s alpha exceeded 0.81 on all dimensions.

These experiments are not intended to compare model performance, but rather to validate the dataset’s difficulty and utility as a benchmark for figurative and culturally grounded generation. Detailed scores and examples are provided in the Supplementary Material.

All code and scripts used for model training, clustering, and evaluation are publicly available (see Code Availability), enabling full reproducibility of this technical validation.

## Supplementary information

Supplementary Information

Supplementary Data 1

Supplementary Data 2

## Data Availability

The derived dataset described in this article is publicly available in a permanent, DOI-backed repository. The dataset consists of a single UTF-8 encoded JSON file containing 5,252 Yemeni proverbs with associated explanations and metadata fields. Due to licensing considerations, the original source texts from printed books and online platforms are not redistributed. Only derived data are shared. Users wishing to access the original materials must obtain them directly from the original sources under their respective terms of use. The dataset is released under a Creative Commons Attribution 4.0 International (CC BY 4.0) license, which applies only to the derived data provided with this publication. The dataset can be accessed at: 10.5281/zenodo.18813628.

## Code availability

All code used for data extraction, cleaning, normalization, validation, analysis, and model-based evaluation is publicly available. This includes scripts for web scraping, preprocessing, dataset consolidation, thematic clustering, model fine-tuning, and automatic evaluation.

The codebase is hosted in a public GitHub repository at: https://github.com/NasserThmer/Yemeni-Proverbs-dataset-code.

The repository contains a detailed README file with instructions for reproducing all processing and analysis steps described in this manuscript.
