# Supplementary material for: A Benchmark Corpus of Yemeni Proverbs for Figurative and Cultural Language Modeling
Source: Sci Data. 2026 May 11;13:1073. doi: 10.1038/s41597-026-07234-y (PMC13381537; doi:10.1038/s41597-026-07234-y)
Supplement: Supplementary file 1 — Supplementary Information [file 41597_2026_7234_MOESM1_ESM.pdf]

Supplementary Information

A Benchmark Corpus of Yemeni Proverbs for Figurative and Cultural Language Modeling

Contents

Section	Title
1	Supplementary evaluation overview
2	Automatic evaluation results for zero-shot prompting
3	Automatic evaluation results for few-shot prompting
4	Inter-annotator agreement
5	Supplementary data workbooks

1. Supplementary Evaluation Overview

This supplementary information reports additional evaluation results for seven large language models evaluated on Yemeni proverb explanation generation under zero-shot and few-shot prompting settings. Automatic evaluation was conducted using Cosine Similarity, BERTScore-F1, and Semantic Answer Similarity (SAS). Inter-annotator agreement for the human evaluation was measured using Krippendorff's alpha across three criteria: semantic accuracy, cultural appropriateness, and clarity.

The full per-example automatic evaluation outputs are provided in the accompanying Excel workbooks listed in Section 5.

2. Automatic Evaluation Results for Zero-shot Prompting

Table S1. Zero-shot automatic evaluation results averaged over 456 proverb examples.

Model	Rows	Cosine MiniLM	Cosine CAMeL	Cosine Arabic-KW	SAS	BERTScore-F1
DeepSeek-LLM-7B-Chat	456	0.7686	0.8244	0.3454	0.2165	0.6854
ALLaM-7B-Instruct-Preview	456	0.7641	0.8494	0.5409	0.3793	0.7272
Jais-Adapted-13B-Chat	456	0.6169	0.7985	0.4702	0.3005	0.6709
Mistral-7B-Instruct-v0.2	456	0.6622	0.7305	0.2601	0.1287	0.6495
Gemini 1.5 Pro	456	0.7752	0.8414	0.5676	0.4623	0.7257
GPT-4o	456	0.7887	0.8691	0.6238	0.5079	0.7357
Meta-Llama-3-8B-Instruct	456	0.7285	0.8470	0.5423	0.3734	0.7050
**Average**	456	0.7292	0.8229	0.4786	0.3384	0.6999

3. Automatic Evaluation Results for Few-shot Prompting

Table S2. Few-shot automatic evaluation results averaged over 456 proverb examples.

Model	Rows	Cosine MiniLM	Cosine CAMeL	Cosine Arabic-KW	SAS	BERTScore-F1
ALLaM-7B-Instruct-Preview	456	0.8693	0.8904	0.6829	0.5279	0.7891
DeepSeek-LLM-7B-Chat	456	0.8465	0.8477	0.5517	0.3651	0.7674
Jais-Adapted-13B-Chat	456	0.4014	0.6871	0.1265	0.1768	0.6071
Mistral-7B-Instruct-v0.2	456	0.8547	0.8471	0.5893	0.4070	0.7711
Gemini 1.5 Pro	456	0.7974	0.8489	0.6247	0.5190	0.7397
GPT-4o	456	0.7653	0.8653	0.6672	0.5780	0.7534
Meta-Llama-3-8B-Instruct	456	0.7996	0.8230	0.5855	0.4220	0.7362
**Average**	456	0.7620	0.8299	0.5468	0.4280	0.7377

4. Inter-Annotator Agreement

Table S3. Average Krippendorff's alpha across the seven evaluated models.

Setting	Semantic accuracy	Cultural appropriateness	Clarity	Mean alpha
Zero-shot	0.8380	0.8350	0.8380	0.8370
Few-shot	0.8440	0.8510	0.8440	0.8460
**Overall average**	0.8410	0.8430	0.8410	0.8420

Krippendorff's alpha exceeded 0.81 for all three evaluation dimensions in both prompting settings.

5. Supplementary Data Workbooks

Table S4. Supplementary data files accompanying this supplementary information.

Supplementary file	Description	Workbook contents
Supplementary_Data_Zero_Shot.xlsx	Detailed zero-shot automatic evaluation results for seven models.	One Summary sheet and one sheet per model, with proverb text, reference explanation, generated explanation, Cosine MiniLM, Cosine CAMeL, Cosine Arabic-KW, SAS, and BERTScore-F1.
Supplementary_Data_Few_Shot.xlsx	Detailed few-shot automatic evaluation results for seven models.	One Summary sheet and one sheet per model, with proverb text, reference explanation, generated explanation, Cosine MiniLM, Cosine CAMeL, Cosine Arabic-KW, SAS, and BERTScore-F1.
